# Modern Sociology

**Published:** 1897-01-09

**Authors:** 


### Modern Sociology.

### THE COMPETITION OF THE CONVICT.

In the recent agitation against the importation of foreign goods at lower prices than similar things could be made for in England, a special point has been made of the injury done to the British workman by the use of "foreign prison-made goods." The implication is that prison-made articles, because the workmen receive no wage, can be sold at a price at which no free workman can produce them and live. Such an assertion is worth inquiring into. If true, it will certainly betray a cruel injustice.

Unfortunately, the people who are most ready to make the assertion seem the least willing to support it by statistics, and we are forced to rely on our own investigations. The United States, more systematically than any other civilised State, makes inquiry into such questions; and it is possible that, in a land where every State is the size of a kingdom, we may find means of comparison as good as in the countries of Europe. In America, convicts are employed in various ways. In some States they labour at public works, and that, where practicable, is the best form of labour. We must keep criminals out of mischief, and we are, as a nation, honestly willing to pay for so keeping them; but, at the same time, we do not want to pay more than is just and necessary, and we are averse, on both moral and economical grounds, from keeping any creature in idleness for months or years. Penal servitude should mean penal work, or we may find our convict a lunatic at the end of his "time"—or, at best, a man sent out into the world as unfit to earn an honest living as when he entered the prison gates.

The prime justification of convict labour is that it gives the convict power to become an honest man. Every other consideration must be secondary to this. To teach a trade to a man who hitherto has stolen, forged, or cheated for a living is to put in his hands a weapon he has never yet used. That this is the result of such training on those who have fallen but once is proved by the experience of the Elmira Reformatory, and his would be an unworthy soul who should say that the money spent in fitting a man to take his real place in life is ill-spent, though it should never be refunded.

In fact, the notion of making a criminal a paying investment is wrong. If he earns more than the cost of his keep, the surplus should, we think, be put away to give into his hands the day he regains liberty. But does he indeed earn money for the State that confines him? Study the average criminal; one thing he knows nothing of—daily, regular, steady work. He may, after a fashion, be kept at work by compulsion, but this means little. It has been proved that the labour of the slave can in no degree compare in efficiency with that of the free man; this is true in an even higher degree of the convict. He does not know the trade he is set to, and he does not want to learn it. He is as idle as his taskmaster will permit; he makes mistakes which a man who felt that his post in the factory was at stake would never fall into. A severe discipline may make him do something, but it certainly has not the inspiring power that dwells in the independent workman. If, therefore, the independent workman cannot make a living out of his work, the convict product will be less valuable still. Speaking generally, the convict is not self-supporting. Of course, on the principle that every little helps, his masters may be glad to sell his produce for anything it will fetch, but it will soon become obvious that there is very little profit in this, and the business will not be energetically pursued. In the United States, there has been in most of the States and territories a decrease in the value of the work during the last ten years. In some cases, it must be admitted, this is due to the passing of State laws intended to prevent the prisons competing with free labour. But these are a small minority. In several prisons it was found that it was impossible to compete with those doing the same work outside. Not only were the outside workers more active, but the outside factories were prepared to go to expense in the erection of the latest fashions in machinery which the prison authorities could not face. The low selling value of the goods produced is stated in a large number of cases to be the reason of an industry ceasing to become remunerative, and being given up.

In this case the prison was the employer of labour, and suffered from the fluctuations of the market. This is known as the public-account system, and, when at all profitable, usually pays the State better than any other method; but it seems to be admitted that it cannot generally compete with free labour.

Other methods of employing convicts are all variations of the contract system. Sometimes a contractor employs prisoners at a fixed price per day, and the prison furnishes therewith the mechanical power and sometimes the machinery for carrying on the work. This is, we should think, a very convenient plan for the contractor; whether it is judicious for the State to provide so much for a private enterprise is another question. Another form is what is called the "piece price" system, by which the manufacturer provides the materials of the chosen industry in a form ready for working up, and buys back the finished products at a fixed price. The management of work and workers is, however, left to the prison officials. The "lease" system, on the other hand, hires out convicts for a given time to a contractor, who clothes and feeds them, and on whom is laid the onus of maintaining discipline among his convict servants. We think this last system is fundamentally bad. When the State takes charge of a criminal it does so primarily for purposes of discipline, and this duty it has no right to delegate. Under the lease system there is no security against brutality being shown towards the convicts, or, on the other hand, against the most demoralising relaxation of discipline.

Indeed, we think the exploiting of convict labour reprehensible. Prison work should be primarily educative. Any labour beyond this should be as far as possible devoted to work intended for use in the prison, to repairs, cleansing, &c. In the third place should come work on objects of public utility. Only in the last place should any industry be pursued that will compete with free labour, and, if followed, due allowance should be made for the cost and upkeep of machinery, for the wages of supervision, and for the maintenance of the workers. And if the due proportion of such expenses is added to the selling price of the product, we do not think free labour has much to fear. In fact, there is more likelihood, as the authorities of many American prisons have found, that the workshops will not pay.

